# A longitudinal characterization of the Non-Cystic Fibrosis Bronchiectasis airway microbiome

**DOI:** 10.1038/s41598-019-42862-y

**Published:** 2019-05-03

**Authors:** T. E. Woo, R. Lim, A. A. Heirali, N. Acosta, H. R. Rabin, C. H. Mody, R. Somayaji, M. G. Surette, C. D. Sibley, D. G. Storey, M. D. Parkins

**Affiliations:** 1 Department of Biological Sciences, University of Calgary, Calgary, AB Canada; 2 Department of Medicine, University of Calgary, Calgary, AB Canada; 3 Department of Microbiology, Immunology and Infectious Diseases, University of Calgary, Calgary, AB Canada; 4 Departments of Medicine, and Biochemistry and Biomedical Sciences, McMaster University, Hamilton, Ontario Canada

**Keywords:** Infectious-disease diagnostics, Microbiome

## Abstract

A diverse microbiota exists within the airways of individuals with non-cystic fibrosis bronchiectasis (nCFB). How the lung microbiome evolves over time, and whether changes within the microbiome correlate with future disease progression is not yet known. We assessed the microbial community structure of 133 serial sputa and subsequent disease course of 29 nCFB patients collected over a span of 4–16 years using 16S rRNA paired-end sequencing. Interestingly, no significant shifts in the microbial community of individuals were observed during extended follow-up suggesting the microbiome remains relatively stable over prolonged periods. Samples that were *Pseudomonas aeruginosa* culture positive displayed markedly different microbial community structures compared to those that were positive for *Haemophilus influenzae*. Importantly, patients with sputum of lower microbial community diversity were more likely to experience subsequent lung function decline as defined by annual change in ≥−1 FEV_1_% predicted. Shannon diversity values <1 were more prevalent in patients with FEV_1_ decline (P = 0.002). However, the relative abundance of particular core microbiota constituents did not associate with risk of decline. Here we present data confirming that the microbiome of nCFB individuals is generally stable, and that microbiome-based measurements may have a prognostic role as biomarkers for nCFB.

## Introduction

Bronchiectasis manifests as the abnormal dilation and thickening of the airways secondary to impaired mucocilliary clearance^1^. Studies of bronchiectasis have focused on infections caused by *Pseudomonas aeruginosa* and *Haemophilus influenzae* due to the prevalence of these pathogens in cultured respiratory samples, relationship with accelerated lung function decline, increased exacerbation frequency, and associated morbidity and mortality^2,3^. More recently, however, our understanding of chronic respiratory infections has evolved from a one-host-one-pathogen model to a polymicrobial model of infection^4^. A community of microorganisms, including bacteria, fungi, and viruses, collectively refered to as the lung microbiome, have been demonstrated in airways^5–7^. However, the role these organisms play in health and disease is not well understood^8,9^.

Our knowledge of infections in non-cystic fibrosis bronchiectasis (nCFB) lags behind that in cystic fibrosis (CF). Whereas the lung microbiome of paediatric CF patients is dynamic and changes throughout their adolescence^10,11^, by adulthood a core climax community evolves which remains relatively stable thereafter^11–13^. The observation that microbial diversity within the airways of CF patients inversely correlates with lung function in cross-sectional studies suggests that these communities may influence disease progression^6,9,13^. However, how the microbiota changes over time and associates with disease progression remains to be elucidated. With this study, we sought to characterize the natural history of airways communities within individuals with nCFB using a longitudinal collection of samples. Furthermore, we sought to determine if the nCFB microbiota correlated with subsequent long-term pulmonary outcomes and might thus serve as a novel biomarker to predict future clinical course.

## Results

### Patient Demographics

One hundred and thirty-three sputum samples from 29 patients were included. Demographics and baseline patient characteristics are listed in Table 1. The median duration of follow-up was 5.8 years (IQR 5.0–7.9, range 4.3–16). The majority of patients were never-smokers (93%; 27/29). Etiologies of bronchiectasis included post-infectious (62%), idiopathic (34%) and ciliary dyskinesia (4%). Lung function was generally stable during follow up with a median change in FEV_1_% percent predicted of −0.29%/year (IQR: −1.3%–+0.65%). Twelve exacerbation events were captured in the selected sputa, from 10 of the 29 (34%) patients. Two patients ultimately died and no patients received lung transplantation during the study period.

**Table 1:** Demographics and treatments used in nCFB patients at enrolment.

Patient Demographics (n = 29)	Decliners (n = 11)	Stables (n = 18)	Total (n = 29)
Median Age at Study Enrolment (IQR)	63 (39–70)	57 (50–68)	60 (48–69)
Median FEV_1_ at enrolment (% predicted) (IQR)	71 (45–93)	53 (39–67)	55% (40.5–74)
**Respiratory Comorbidities**			
Sinusitis	5 (45%)	10 (56%)	15 (52%)
Asthma	1 (9%)	3 (17%)	4 (14%)
Chronic Obstructive Pulmonary Disease (COPD)	0	2 (11%)	2 (7%)
**Treatments Recorded During Sample Collection**			
Inhaled corticosteroids	7 (64%)	14 (78%)	21 (72%)
Long-acting beta-agonists (LABA)	6 (55%)	14 (78%)	20 (69%)
Long-acting muscarinic antagonist (LAMA)	1 (9%)	8 (44%)	9 (31%)
Short-acting beta-agonists (SABA)	6 (55%)	12 (67%)	18 (62%)
Ipratropium bromide	3 (27%)	7 (39%)	10 (34%)
Saline	2 (18%)	4 (22%)	6 (21%)
Proton-pump inhibitors	1 (9%)	2 (11%)	3 (10%)
H2-receptor antagonists	1 (9%)	1 (6%)	2 (7%)
**Cultured Pathogens**			
*Pseudomonas aeruginosa*	6 (55%)	10 (56%)	16 (55%)
*Haemophilus influenzae*	4 (36%)	4 (22%)	8 (28%)
*Staphylococcus aureus*	3 (27%)	5 (28%)	8 (28%)
*Moraxella catarrhalis*	2 (18%)	1 (6%)	3 (10%)
*Streptococcus pneumoniae*	1 (9%)	3 (17%)	4 (14%)

Inter-quartile Range (IQR) and percent are indicated by the parenthesis.

### The nCFB Microbial Community Displays Inter- and Intra-patient Variability

**Figure 1:**
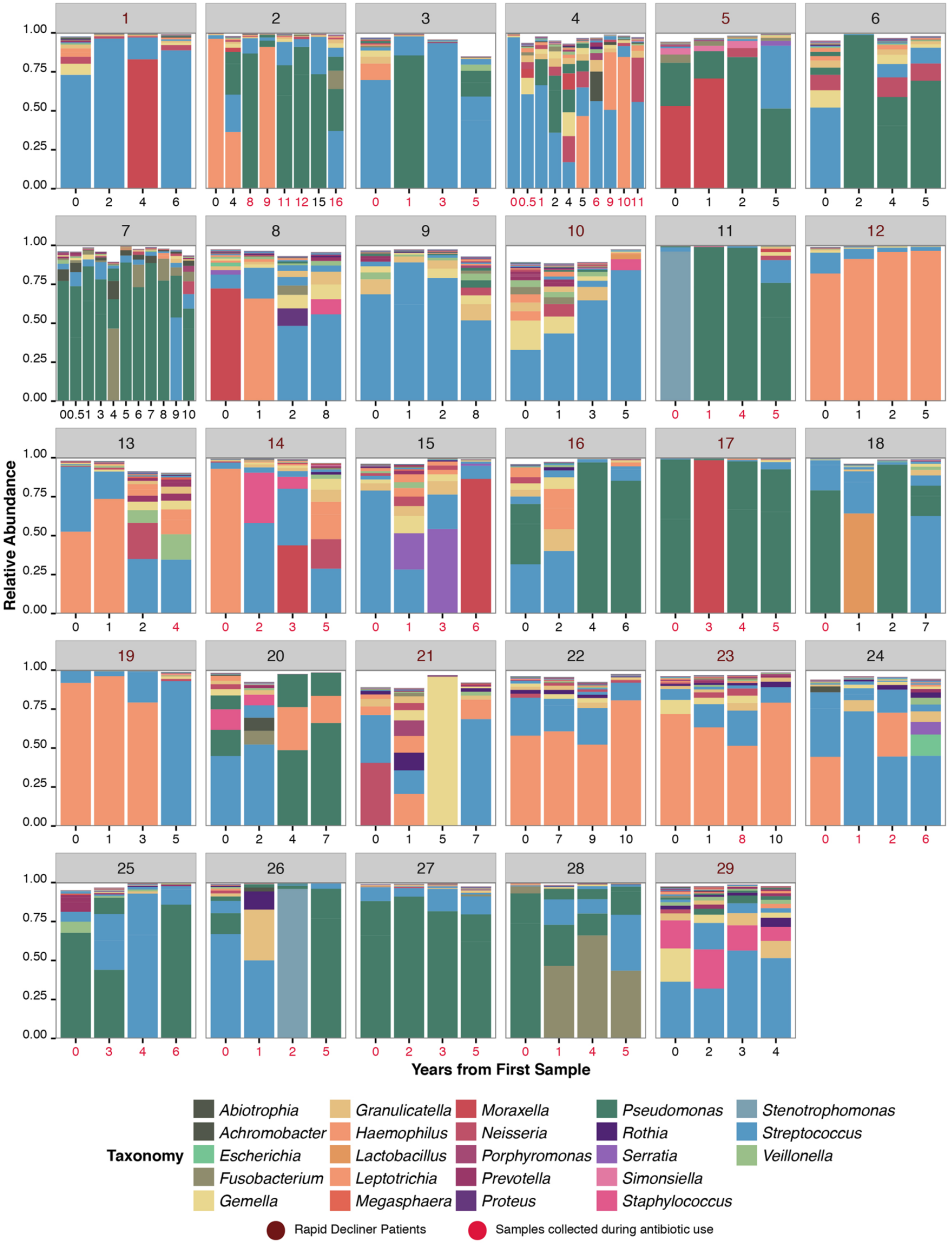
Taxonomic summaries of OTU present in >1% relative abundance of all samples (n = 133) obtained from a cohort of 29 nCFB patients as a function of the years from first sample. Samples for each patient are grouped together by a unique patient identifier – as indicated by the grey heading. Patient’s numbers from the Decliner group are indicated in magenta. Analysis of the taxonomic summary reveals a unique microbiome associated with each patient and varying degrees of inter and intra-patient diversity. Samples collected under antibiotic pressure are indicated by red text indidcating year of collection.

**Figure 2:**
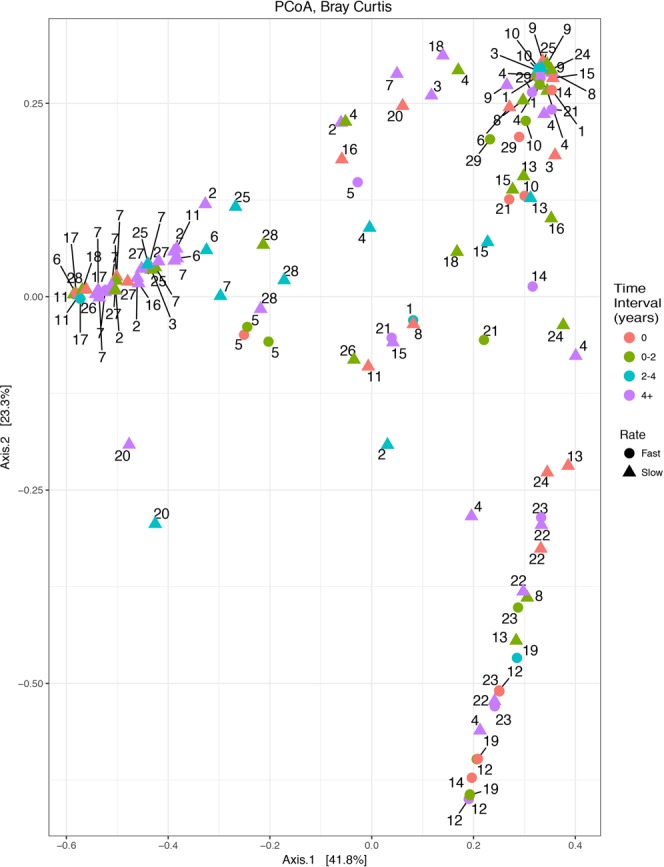
Clustering of samples by patient during stable periods (excluding exacerbation events) was observed (n = 121). Samples obtained from each patient are designated by the patient number adjacent to each point. Samples were categorized by color based on the time since baseline sample. Patients classified as Decliners are indicated by a circle, whereas patients with a Stable course are represented by triangles. Significant clustering was observed using PERMANOVA analysis (p = 0.03).

**Figure 3:**
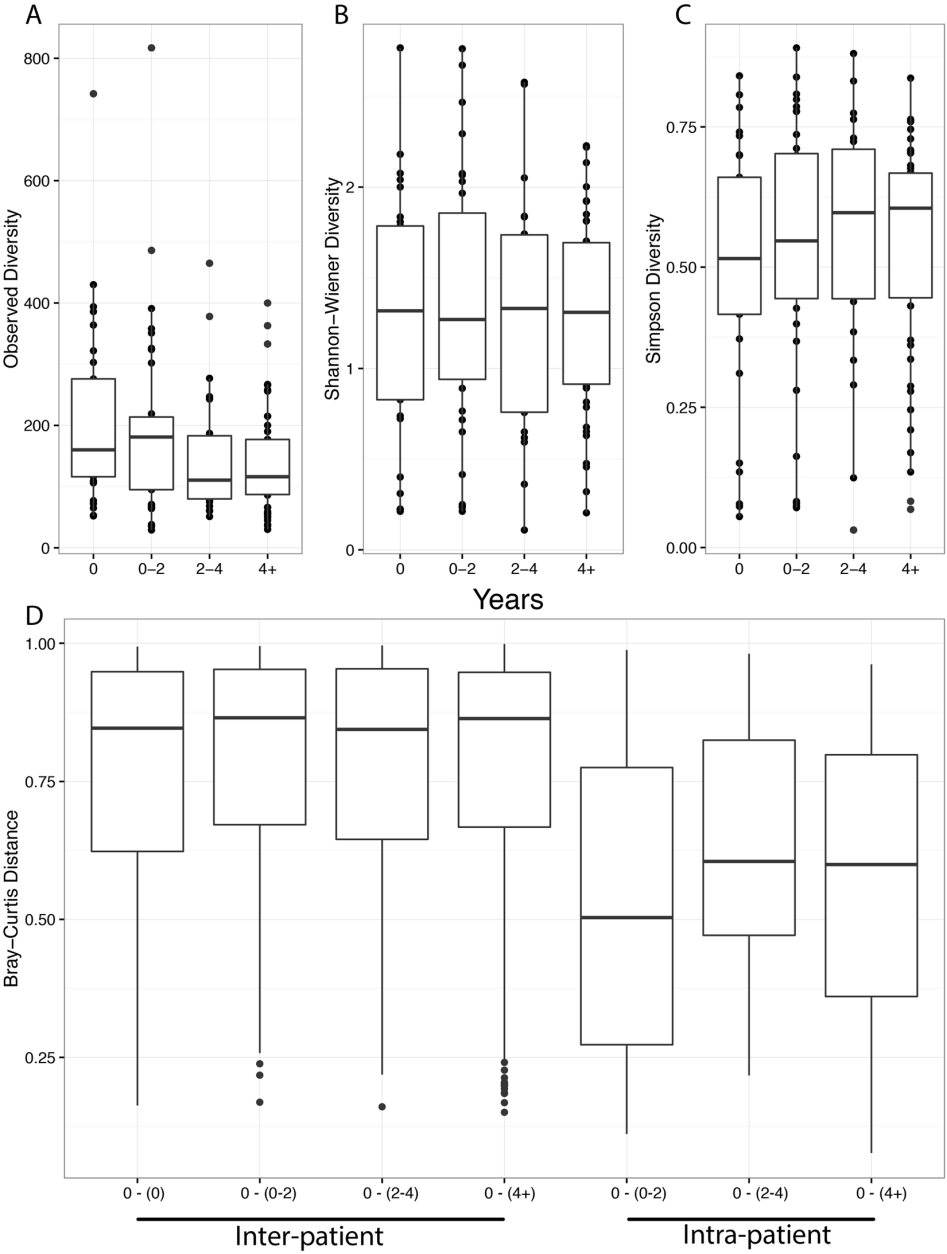
The microbial community was observed to remain relatively constant over the study period as measured by Observed OTUs (**A**), Shannon-Wiener (**B**) and Simpson diversity (**C**), and Bray-Curtis dissimilarity (**D**). Samples were categorized based on the time interval relative to first sample as: 0 (n = 29), 0–2 years (n = 36), 2–4 (n = 22), and 4 + (n = 46) years since initial sample collection. The median and interquartile ranges (IQR) are represented by the middle, top, and bottom lines of each boxplot. No statistically significant comparisons were observed.

A total of 7,698,972 sequences were generated (median of 49,437 sequences/sample, IQR: 33,655–73,464, range: 5136–170,812) with 3202 total observed OTUs (median of 138 OTU per sample, IQR: 90–215, range: 29–817). Across the sample population, the majority of OTUs were classified as *Streptococcus* (30%), *Pseudomonas* (28%), and *Haemophilus* (17%). The longitudinal lung microbiota of taxa found in >1% relative abundance in the dataset was visualized using taxonomic summaries (Fig. 1). Notably, we observed varying degrees of inter- and intra-patient heterogeneity. Whereas patients 22, 23 and 27 had remarkably stable lung microbiomes, patients 4, 11, 15 and 26 demonstrated considerable temporal variation. When samples collected during exacerbations were excluded, significant clustering of samples by patient was observed (PERMANOVA, p = 0.03) (Fig. 2). In order to understand the natural history of the nCFB airways microbiota, we compared the composition of samples by first binning them into distinct time intervals (Fig. 3A–C). No significant changes in alpha-diversity was observed in any of these periods relative to the initial samples and PERMANOVA analysis did not reveal any community-wide differences (p = 0.54). In particular, Bray-Curtis analysis did not reveal any significant inter- and intra-patient changes between the initial, intermediate or final sample intervals - although certain individuals had dynamic ranges (Fig. 3D).

### The nCFB microbiome associates with disease trajectory

**Figure 4:**
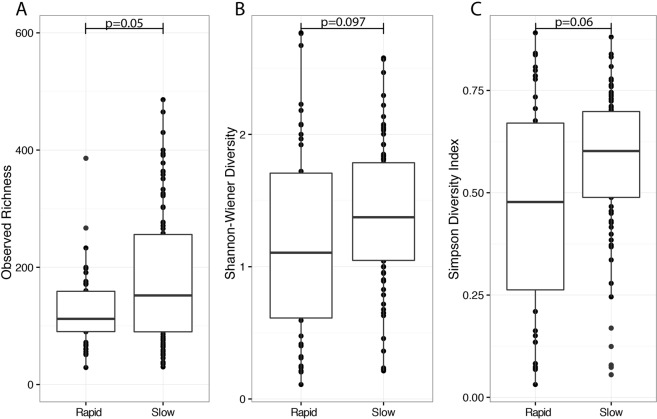
Sputum samples collected from patients (n = 44 samples, 11 patients) who were categorized as Decliners (≥1 FEV_1_% per year) generally displayed lower levels of alpha diversity as compared to Stables (n = 89, 18 patients). No significant differences were detected using the Observed OTU (**A**), Shannon (**B**), and Simpson (**C**) diversity indices. The median and interquartile ranges of each group are represented by the middle, top, and bottom lines of each boxplot. Statistical analysis was performed using the Wilcoxon-signed rank test.

We investigated whether the baseline microbiota - as established from the first sputum sample on record - correlated with either baseline disease severity or subsequent lung function decline using our prospective collection. Notably, baseline demographics were not significantly associated with changes in microbial diversity (data not shown). In our small sample population, we did not confirm that disease severity at baseline associated with either alpha or beta-diversity (Fig. S1). We did, however, observe that baseline microbial diversity correlated with subsequent rate of lung function decline (Fig. 4). Eleven (38%) patients met our *a priori* definition for Decliner (>−1%/year FEV_1_). Median rates of annual FEV_1_ decline between Decliners were −1.8% (IQR: −1.3–−1) vs. +0.4% (IQR: −0.07–1.25) in Stable patients (p = 0.004), respectively. Baseline measurements of Observed OTU (p = 0.90), Shannon diversity (p = 0.51), or Simpson diversity (p = 0.64) were not significantly different between both groups. When comparing these two groups, we identified that Decliners generally had lower alpha-diversity using the Observed OTU (p = 0.05), but not Shannon (p = 0.097) and Simpson (p = 0.06) indices although similar trends were evident. When rarefied, significant differences in Shannon (p = 0.035) and Simpson diversity (p = 0.033), but not Observed OTUs (p = 0.07) were observed. However, no community-wide differences were observed between the two patient groups (p = 1.0). When using alternate definitions for Decliners of −0.5%/year and −1.5%/year the same trends were evident, but did not reach significance (data not shown).

As lower microbial diversity was observed to associate with Decliner status, we investigated whether the initial Observed OTU, Simpson, and Shannon diversity measurement was correlated with change in lung function. No significant correlation between the initial diversity measures for each patient and annual rate of lung function decline was found using a generalized linear model using the Shannon (p = 0.61), Observed (p = 0.23) and Simpson diversity (p = 0.61) indices (Fig. S2). Likewise, we found that no association between the abundance of specific genera (found within the overall community at >1% of reads) and subsequent lung function decline. The initial abundance (t = 0) of *Pseudomonas* (*r*_s_ = −0.21), *Haemophilus* (*r*_s_ = 0.34), *Streptococcus* (*r*_s_ = −0.18), *Prevotella* (*r*_s_ = −0.29), *Fusobacterium* (*r*_s_ = −0.18), *Gemella* (*r*_s_ = −0.10), *Granicutella* (*r*_s_ = −0.06), *Neisseria* (*r*_s_ = 0.08), *Rothia* (*r*_s_ = −0.08), and *Staphyloccocus* (*r*_s_ = 0.17) within each sample did not correlate strongly with lung function for each patient. Lastly, we examined whether Shannon diversity measurements associated with the clinical prognosis of patients within our study. Using a Shannon diversity index cut-off value of 1, used for biomarker studies in CF^14^, we observed that the majority of samples from Stable patients had a Shannon diversity value of >1 (73/93 samples) as compared to Decliners (20/40 samples) (p = 0.002). When the analysis was constrained by patient, similar trends were observed but did not reach significance (p = 0.39). Samples with a Shannon diversity index <1 were associated with changes in the microbial community (p = 0.04). In particular the abundance of seven genera had relative lower abundance; *Gemella*, *Lactobacillus*, *Nicoletella*, *Haemophilus*, *Bordetella*, *Staphylococcus*, and *Achromobacter* (Fig. S3) whereas *Streptococcus* and *Moraxella* were increased.

### *Pseudomonas* and *Haemophilus* display an antagonistic relationship in nCFB airways

**Figure 5:**
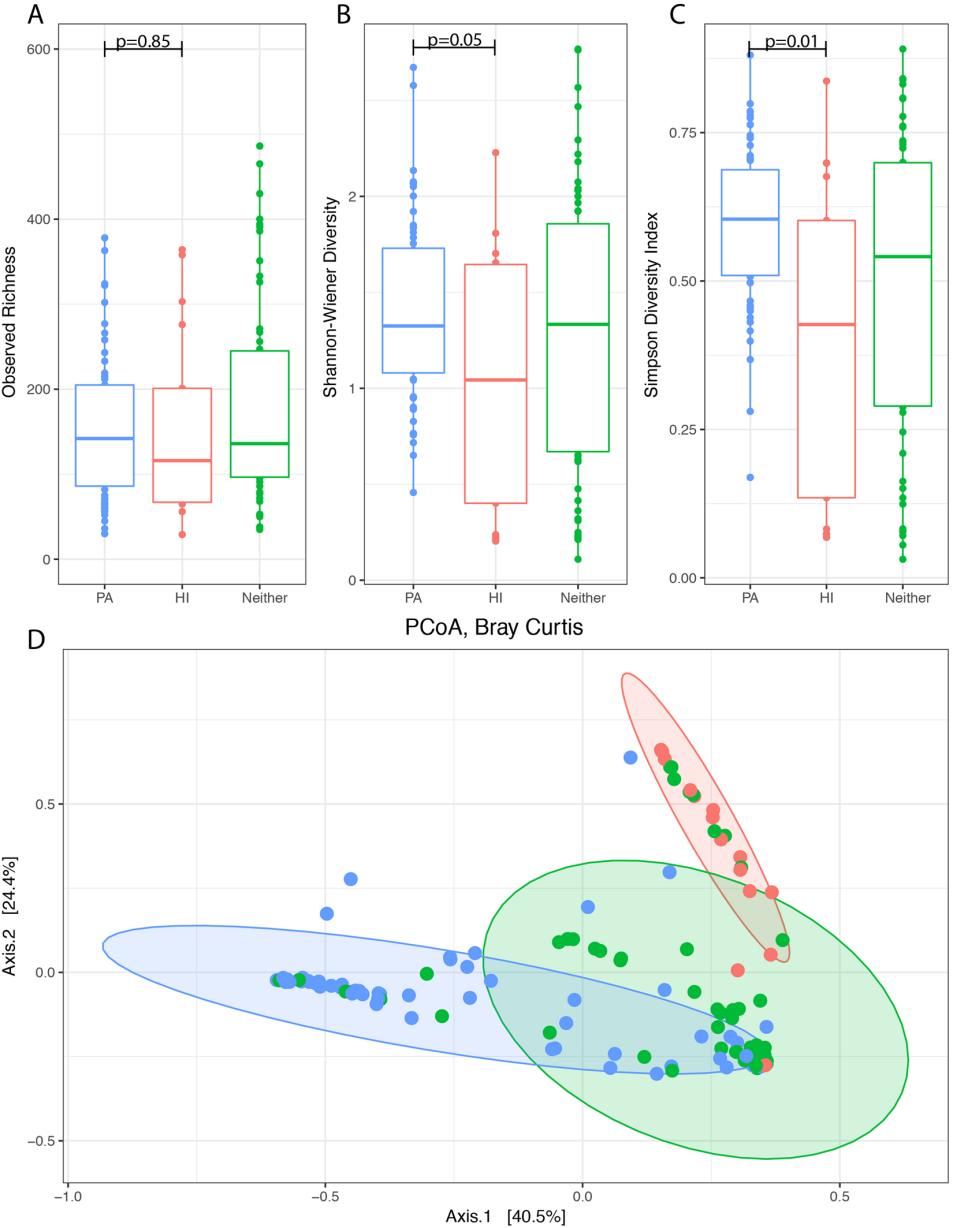
Observed OTU (**A**), Shannon (**B**), and Simpson (**C**) diversity, and Bray-Curtis dissimilarity (**D**) illustrate clustering of *P*. *aeruginosa* and *H*. *influenzae* positive samples. Differences in the sputum microbial diversity of samples collected in relation to the growth of *Pseudomonas aeruginosa* (PA, n = 60) and *Haemophilus influenzae* (HI, n = 17) and in samples with neither (Neither, n = 56). The ellipses for each group represent the 90% confidence interval. The median and interquartile ranges (IQR) are represented by the middle, top, and bottom lines of each boxplot. Statistically significant comparisons were shown using the p-value.

We observed a strong correlation between airway pathogens obtained during routine real-time clinical laboratory culture and microbial community diversity determined retrospectively from these same samples. *P*. *aeruginosa* was isolated in 60 (45%) of the total samples (16/29 patients), whereas *H*. *influenzae* was only found in 17 samples (13%) (8/29 patients). Samples which were culture positive for *P*. *aeruginosa* trended towards coming from individuals with worse baseline lung disease relative to *H*. *influenzae* positive individuals (median FEV_1_ of 53% (IQR: 36–62) versus 65% (IQR: 39–75) (p = 0.10). We observed that the real-time isolation of *P*. *aeruginosa* was exclusive to *H*. *influenzae* (and vice-versa) such that these two organisms were never recovered together in culture from the same sample. Further differences arose when assessing the community microbiota in samples obtained in association with *P*. *aeruginosa*, *H*. *influenzae*, and in samples with neither (Fig. 5). When comparing these three groups, we found that *P*. *aeruginosa* culture positive samples had relatively higher Simpson (p = 0.01) and Shannon (p = 0.05) diversity, but not Observed OTU diversity (p = 0.85) as compared to *H*. *influenzae* culture positive samples. No significant differences in the alpha diversity were observed between either *P*. *aeruginosa* or *H*. *influenzae* culture positive samples and samples where neither of these organisms were identified. Community-wide differences were observed using PERMANOVA analysis (p = 0.001). In particular, the relative abundance of 10 genera was found to be significantly different between samples (Fig. S4). Interestingly, the increased log-fold relative abundance of *Pseudomonas* was observed in tandem with a >5 fold decrease change in relative abundance of *Haemophilus* (Fig. S5).

### Acute antibacterial therapies introduce transient shifts within the microbiome

**Figure 6:**
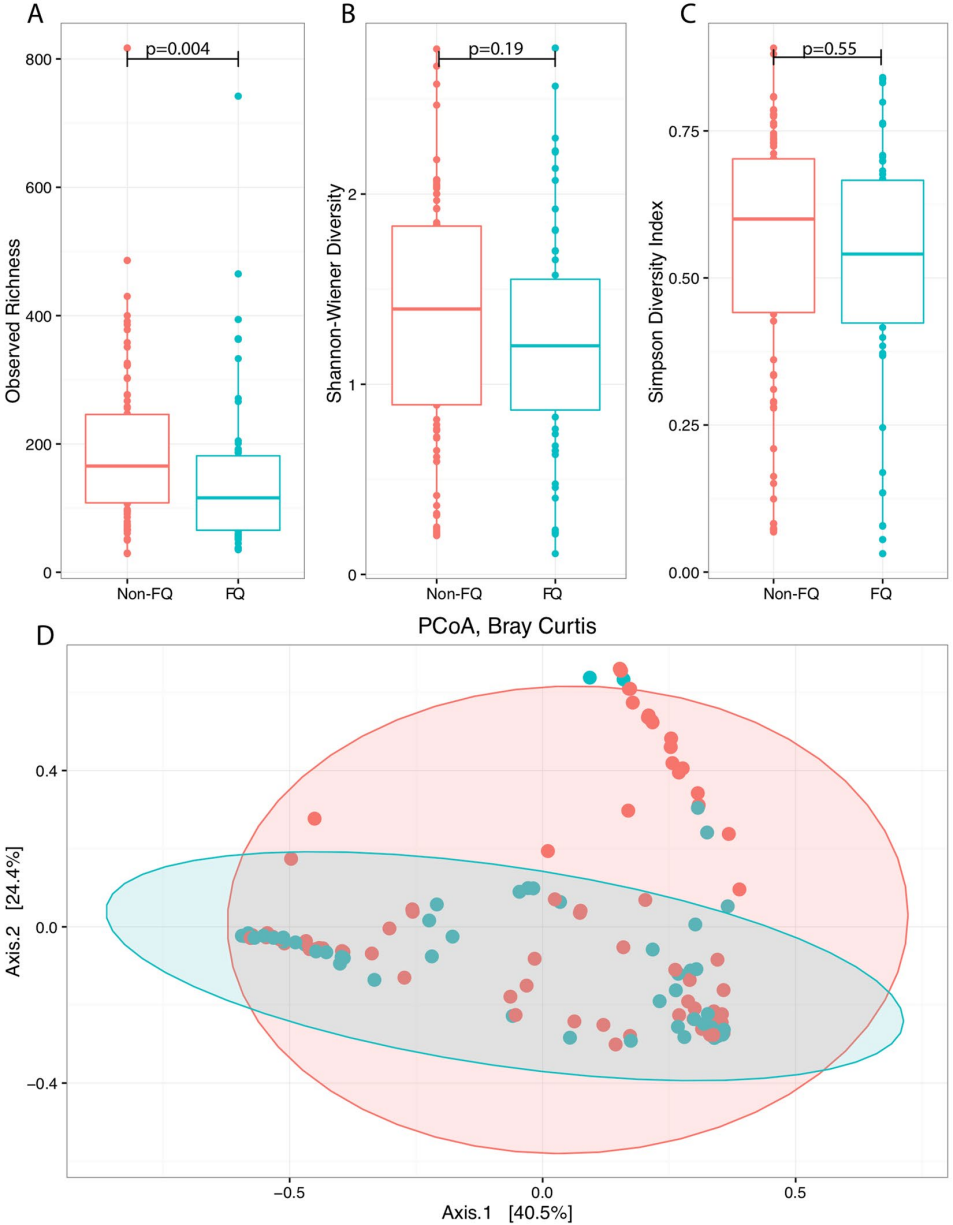
Acute fluoroquinolones use was associated with reduced community richness as represented by the Observed OTU. Samples collected during acute fluoroquinolone use (FQ, n = 55) were compared to samples collected in their absence (No-FQ, n = 78). Significant differences were seen in Observed OTUs (**A**), but not for Shannon (**B**) or Simpson diversity (**C**). Significant community-wide differences were found using PERMANOVA when permutations were constrained by patient and visualized using PCoA (p = 0.01).

When we examined the influence of acute antimicrobial therapy with microbial changes in community structure, only fluoroquinolone use was associated with changes in diversity. Observed OTU richness (p = 0.004) was reduced under acute antibacterial pressures, but not Shannon (p = 0.19) or Simpson (p = 0.55) diversity indices (Fig. 6). Interestingly, community-wide differences were observed using PERMANOVA analysis (p = 0.01). In particular, samples obtained during fluoroquinolone use were found to have significant changes in the abundance of 12 genera. Two of the twelve genera, *Escherichia* and *Moraxella*, had increased relative abundance and the remaining had decreased relative abundance including: *Veillonella*, *Dialister*, *Azorhizophilus*, *Bulleidia*, *Capnocytophaga*, *Lactobacillus*, *Prevotella*, *Treponema*, *Haemophilus*, and *Achromobacter* (Fig. S6).

## Discussion

The lung microbiome has been suggested to play an important role in modifying disease progression for individuals with CF and nCFB^6,11,12^. Whereas recent studies have alluded to the relative stability of the nCFB microbiome over short periods^15^, we believe our ability to assess serial samples collected up to 16 years apart, provides unique insight into the longer-term adaptations of the lung microbiome. Interestingly, we observed no significant changes in the alpha and beta-diversity of the lung microbiome. This is all the more surprising given the extended duration of observation (for some patients up to 16 years). Stability was not influenced by baseline clinical characteristics or disease severity. Indeed, even over protracted periods of time, we observed, as others have over short time periods, microbiota tended to cluster by patient, alluding to unique patterns of colonization^15^. In comparison, studies in CF suggest that the childhood microbiota fluctuates and changes over time, while the adult lung microbiome remains relatively stable^10,12,16^.

Given the limited capacity by which cultured pathogens within nCFB airways predict clinical outcomes, the potential of the microbiome to serve as a biomarker for clinical management is of great interest. For instance, early studies have alluded to the predominance of *P*. *aeruginosa* and *Veillona* species as predictors for future exacerbations^17^. In contrast to early point prevalence studies, we observed no significant relationship between alpha diversity and static measures of lung function^6,18^. However, these trends are not ubiquitous amongst all studies, with other studies showing no association between diversity and lung function^19^. Rather, we observed a general relationship between alpha diversity and the rate at which lung function declined in these patients. It is likely that the slow rate of progressive lung disease was prohibitive in establishing community-wide differences as no significant decline was observed between baseline and end-of-study lung function measures within our cohort. Furthermore, we observed that samples with Shannon diversity values <1 tended to be associated with Decliner status and may warrant further interest in its use as a biomarker.

Amongst the pathogens isolated from the airways, *P*. *aeruginosa* and *H*. *influenzae* play an important role. The presence of these organisms within the airways has been associated with decreased microbial diversity, worsened clinical outcomes, and increased morbidity and mortality^4,11,20,21^. Importantly, the interactions between these airway pathogens is of great interest, with an antagonistic relationship observed in both cultured and culture-independent microbiome^4,8^. Similarly, we believe that these trends may reflect both direct and indirect competition between these two species. It is possible that it is the accessory microbiome associated with *Pseudomonas*-dominant patients that may exclude the colonization of *H*. *influenzae and* vice-versa^8^. Further, this competition may extend to other organisms and may explain the change in genera observed within our study. Though our V3/V4 sequencing of the 16S rRNA allowed only for genus level classification, our complimentary analysis of real-time culture data revealed that the only *Pseudomonas* and *Haemophilus* species identified from these samples were *P*. *aeruginosa* and *H*. *influenzae*.

The impact of acute exacerbations on microbial communities is not well understood – with no significant changes in the microbiome associated with the onset of these events^19^. Antibiotic use is the mainstay of management of exacerbations in patients with CF and nCFB. Indeed, antibiotic therapy may influence the respiratory microbiota and play an important role in clinical prognosis^4,11,20,21^. Within our study and in congruence with prior findings^15^, we suggest that the broad-spectrum effects of fluoroquinolone decrease the overall richness without affecting the evenness of the microbial community.

Importantly, this retrospective study of prospectively collected samples has a number of limitations. While our sample size of 29 patients is limited, it remains comparable in size to similar microbiome studies in nCFB^6,19^, and even longitudinal studies in CF^16,21^. Furthermore, we believe that the repeated longitudinal measurements analysis – assessing a time period vastly greater than prior works - provides unique insight into the natural history of the disease well beyond cross-sectional studies. Notably, as the primary focus of our study was to assess longitudinal changes in the microbial community, our analysis may have been insensitive to the influence of antimicrobial therapies or the incidence of pulmonary exacerbations on microbial diversity – as seen in previous studies^15,16,22,23^. Given our use of “opportunistically collected samples” during clinical encounters, it may be that our analysis is insensitive to detect changes in the microbial diversity prior to the onset of an exacerbation. As such, studies characterizing samples in their longitudinal relation to such acute events are important in highlighting other factors which may lead to onset of exacerbation^21^. Furthermore, as the vast majority of samples were collected during stable periods it is possible that little antibacterial selective pressures persisted to drive changes in the microbial community. Previous studies in CF have observed that microbial diversity did not change significantly during periods of stability^16^, which we suspect also extends to nCFB. Lastly, our collection of samples is derived from a regional referral clinic and patients are not exclusively seen by nCFB clinic providers and therefore may not be all encompassing.

The Calgary nCFB Biobank represents a unique opportunity in which to characterize the longitudinal nCFB lung microbiome. To our knowledge, this study represents the longest examination of the nCFB microbiome to-date suggesting that the lung microbiome is relatively stable over time, and is highly individualized. We suggest that the lung microbial diversity may be an important contributor to clinical course – although this is likely but one of many host, organism/community and environmental factors. Furthermore, we confirmed the reciprocal relationship between *P*. *aeruginosa* and *H*. *influenzae* in nCFB airways^4,8^. This study has provided further insight into the longitudinal microbiome of individuals with nCFB, and suggests that further studies exploring the microbiome’s association with subsequent clinical outcomes are warranted in our attempt to develop biomarkers for patient prognostication.

## Methods

### Patients and samples

The Calgary Bronchiectasis Biobank (1998–current) is a prospectively inventoried collection of sputum obtained from nCFB adults (>18 years) as part as their routine clinical care and stored at −80 °C. Patients followed at the Calgary Bronchiectasis Clinic have symptoms consistent with and radiographic evidence of bronchiectasis^24^. For inclusion in this study patients had to have ≥4 sputum samples spanning ≥4 years and a diagnosis of nCFB. Individuals with bronchiectasis due to CF were excluded.

### Clinical Information and Definitions

Patient demographic and clinical information was collected through detailed chart review. Pathogens detected through traditional culture-based approaches from these samples were reported in real-time from the clinical microbiology laboratory and these data were extracted during chart review. Baseline patient characteristics including bronchiectasis aetiology, gender, respiratory therapies, antibacterial therapies and co-morbidities were analyzed for their relationship to microbial diversity. Bronchiectasis aetiology was classified as post-infectious, idiopathic, or other. Lung disease stages were classified by spirometry as determined using the Knudson calculation as advanced; Forced expiratory volume in one second (FEV_1_) < 40% predicted, moderate; 40–70%, mild; ≥70%^18^. Rates of lung function decline were calculated by dividing the FEV_1_% by the total number of years followed for each patient. Patients with an annual decline in FEV_1_% predicted of ≥1% were *a priori* considered Decliners (D)^25,26^ whereas those whose decline was less than this were considered Stables (S). Samples were separately coded and considered to be collected during acute antimicrobial therapy if obtained within two weeks of a new antibiotic administration (as acute parenteral antibiotic therapies have been associated with transient changes in the microbiota of suppurative lung disease)^27–29^. In order to assess differences in community composition over time, samples were categorized based on years from collection relative to the initial sample (0), >0.25–2 years, >2–4 years, and >4 years for each patient over the study period. The study was were performed in accordance with relevant guidelines and regulations of the University of Calgary and consistent with those required by Scientific Reports. The Conjoint Health Research Board of the University of Calgary has approved the ongoing collection and maintenance of the biobank and the samples maintained in it (REB16-0035). Furthermore, the CHREB has granted the investigators permission to evaluate patient outcomes associated with biobank derived samples REB16-0854.

### DNA Isolation, 16s rRNA Variable 3–4 (V3/V4) Amplification, and Sequence Processing

Genomic DNA was isolated from sputum samples as previously described^30,31^. Barcoded universal primers adapted from Bartam *et al*. were used to amplify the V3 and V4 region of the 16s rRNA prior to MiSeq Illumina sequencing^32^. Reagent blanks were run for each set of DNA extractions and DNA amplification. If contamination was seen in the technical controls, samples were excluded and the DNA extraction and amplification was subsequently repeated. Technical replicates were not sequenced. Sequence analysis was carried out using previously published Perl scripts^33^. Low quality reads and primer sequences were removed using Cutadapt^34^ and paired-end reads were merged using PANDAseq^35^. Operational taxonomic units (OTUs) were then generated using AbundantOTU+ based on ≥97% similarity of sequences and classified taxonomically using the Ribosomal Database Project Classifier and the Greengenes database^36–38^. No samples were excluded based on our *a priori* requirement cut-off of >3500 reads. Bacterial OTU tables were generated by the removal of singletons (sequences with single reads) and non-bacterial OTU’s (Supplementary Table 1). Singletons were removed in an effort to more accurately evaluate community diversity by reducing the impact of base substitutions, low-quality reads, variable read lengths, non-target amplification, and undetected chimeric sequences^39,40^. The removal of singletons, while debatable, has been shown to reduce alpha-diversity but does significantly alter beta-diversity and the removal of singletons likely reflects a more accurate community^39^.

### Microbial Community and Statistical Analysis

Analysis of the resulting OTU tables was performed in R (V 3.99) using the phyloseq package^41^. For alpha-diversity and beta-diversity calculations, we included OTUs that were present in ≥1% of total relative abundance. Alpha-diversity was calculated using Observed OTUs as measure of richness, and Shannon and Simpson’s diversity indices which include richness and evenness were all calcuated^6,42,43^. Unless otherwise stated, all analysis was performed without the use of rarefication as it is suggested to avoid introducing false positives^44^. However, a secondary analysis of alpha-diversity was performed after rarefying the samples to a read depth of 15,000. This analysis was not included within the manuscript unless a discrepancy with the non-rarfied data was observed. If discrepancies arose, both the non-rarefied and rarefied data are presented. Alpha diversity indices were compared using Wilcoxon Signed Rank for paired non-parametric factors, and Mann-Whitney U test for unpaired non-normally distributed variables. Beta-diversity differences in the microbiome were analyzed using Bray-Curtis distances and visualized using principal coordinate analysis. Community-wide OTU level differences were assessed using the permutational multivariate analysis of variance (PERMANOVA) using distance matrices using 999 permutations following proportional normalization of the input data^13,45^. If community-wide differences were identified, OTUs belonging to the same genus were analysed using the DeSeq2 package using the test = “wald” and fitType = “parametric” settings^46^. For multiple testing, we utilized the Benjamin Hochberg multiple test correction. Log abundance plots were visualized using ggplot2^47^. A generalized linear model was constructed in R to examine the relationship between microbial diversity and repeated lung function measurements. Each analyzed variable was tested independently. The correlation between the abundance of individual genera and FEV_1_ was examined using the Spearman rank correlation coefficient (*r*_s_). Analysis of clinical characteristic and dichotomous variables was done using the Chi-Squared test and Fischer’s exact test using Prism 5.0 (GraphPad). Asymmetrically distributed variables were represented using median and interquartile ranges (IQR).

## Supplementary information

Figs S1-S6

Supplementary Table 1

## Data Availability

The data from which this study derives is available for analysis as per Scientific Reports requirements (PRJNA514329).
