# Supplementary material for: A longitudinal characterization of the Non-Cystic Fibrosis Bronchiectasis airway microbiome
Source: Sci Rep. 2019 May 3;9:6871. doi: 10.1038/s41598-019-42862-y (PMC6499777; doi:10.1038/s41598-019-42862-y)
Supplement: Supplementary file 1 — Figs S1-S6 [file 41598_2019_42862_MOESM1_ESM.docx]

**Supplementary Information**

**A longitudinal characterization of the Non-Cystic Fibrosis Bronchiectasis airway microbiome**

**Authors:** Woo, T.E.^1,2^, Lim, R.^2^, Heirali, A.A.^3^, Acosta, N.^3^, Rabin, H.R.^2,3^, Mody, C.H.^2,3^, Somayaji, R.^2,3^, Surette, M.G.^3,4^, Sibley, C.D.^3^, Storey, D.G.^1,3^ and MD Parkins^2,3^*

The sequencing data from this project is available at;

[https://www.ncbi.nlm.nih.gov/bioproject/?term=(PRJNA514329)%20AND%20bioproject_sra[filter]%20NOT%20bioproject_gap[filter](https://www.ncbi.nlm.nih.gov/bioproject/?term=(PRJNA514329)%20AND%20bioproject_sra%5bfilter%5d%20NOT%20bioproject_gap%5bfilter)]

Department of Biological Sciences, University of Calgary, Calgary, AB, CANADA^1^

Department of Medicine, University of Calgary, Calgary, AB, CANADA^2^

Department of Microbiology, Immunology and Infectious Diseases, University of Calgary, Calgary, AB, CANADA^3^

Departments of Medicine, and Biochemistry and Biomedical Sciences, McMaster University, Hamilton, Ontario, Canada^4^


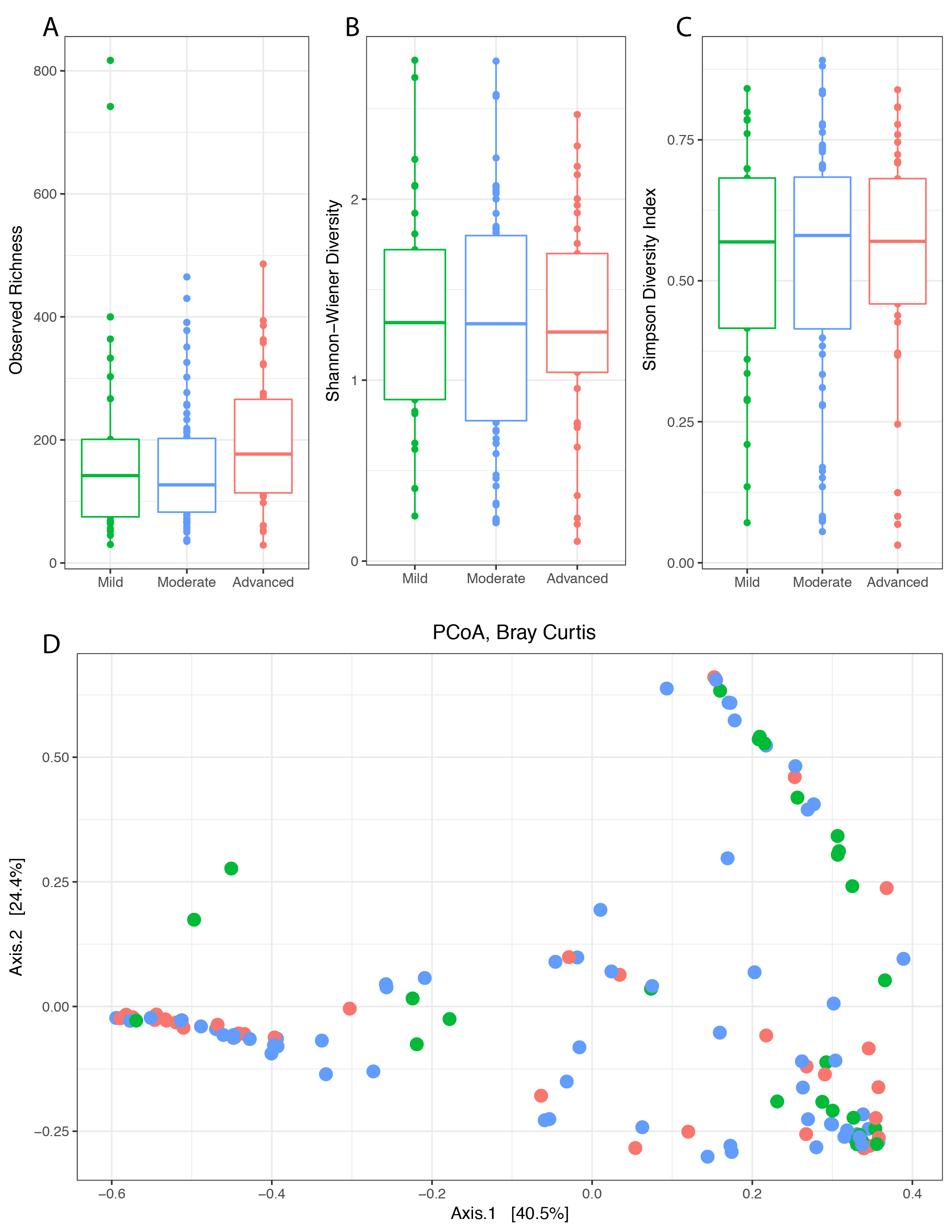


**Figure S1.** Observed Richness (A, p=0.17), Shannon (B, p=0.82), and Simpson diversity index (C, p=0.93), and Bray-Curtis Dissimilarity (D, p=0.97) were observed to remain consistent despite different stages of disease severity. Spirometry was used to determine baseline stage of lung disease which was based on FEV_1_ (%) predicted; mild (n=29): >71, moderate (n=67): 40 – 70%, and advanced (n=37): <40%. The median and interquartile ranges (IQR) are represented by the middle, top, and bottom lines of each boxplot. The median of each group was compared using the Kruskal-Wallis test and deemed significant if p<0.05.

**
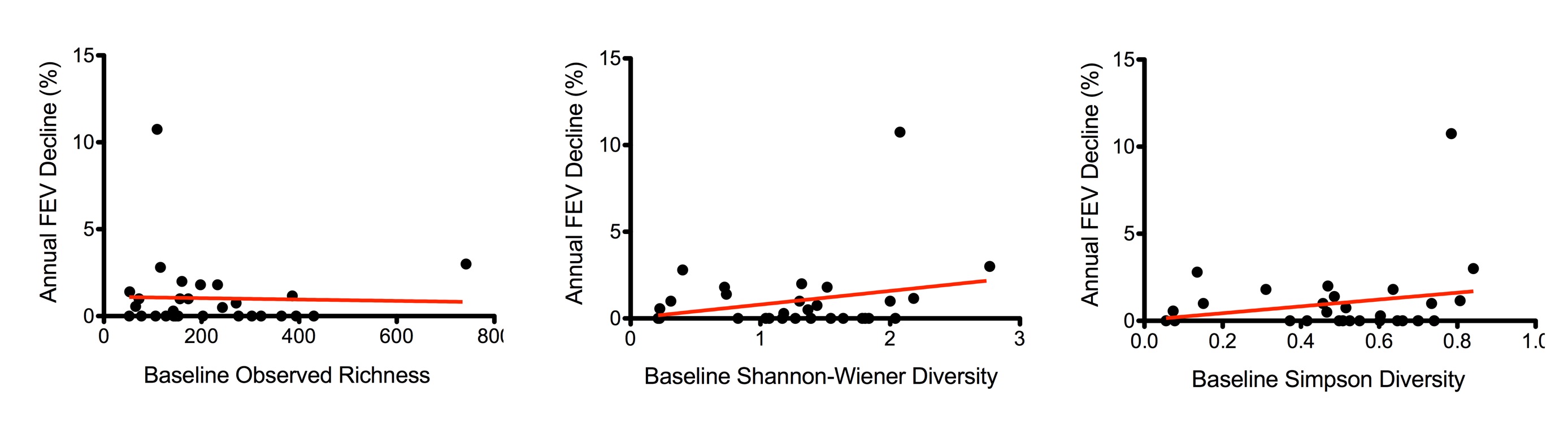
Figure S2.**  **Initial Observed, Shannon-Wiener, and Shannon diversity measures were compared to the rate of annual lung function decline, as measured by FEV_1_ (%) decline per year for all patients (n=29).** No significant correlation between alpha diversity measures and rate of lung function decline was observed. Any patient with no annual decline in FEV1(%) were designated as zero on the graph. Linear regression was performed using Prism and the line of best fit is represented in red.


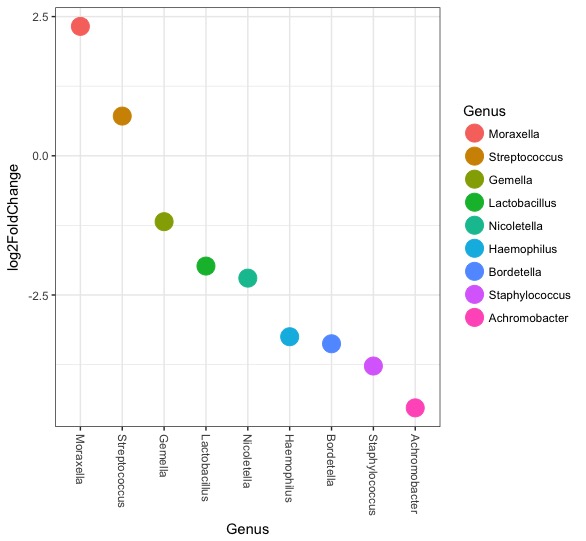


**Figure S3.** **Nine significant genus-level difference were found in samples with a Shannon diversity index <1.** Significance was determined using Bray-Curtis dissimilarity test and were visualized using DeSeq2. The change in log_2_ fold abundance of each genus was deemed significant if α<0.01. OTUs were grouped into genera prior to Beta-diversity analysis.


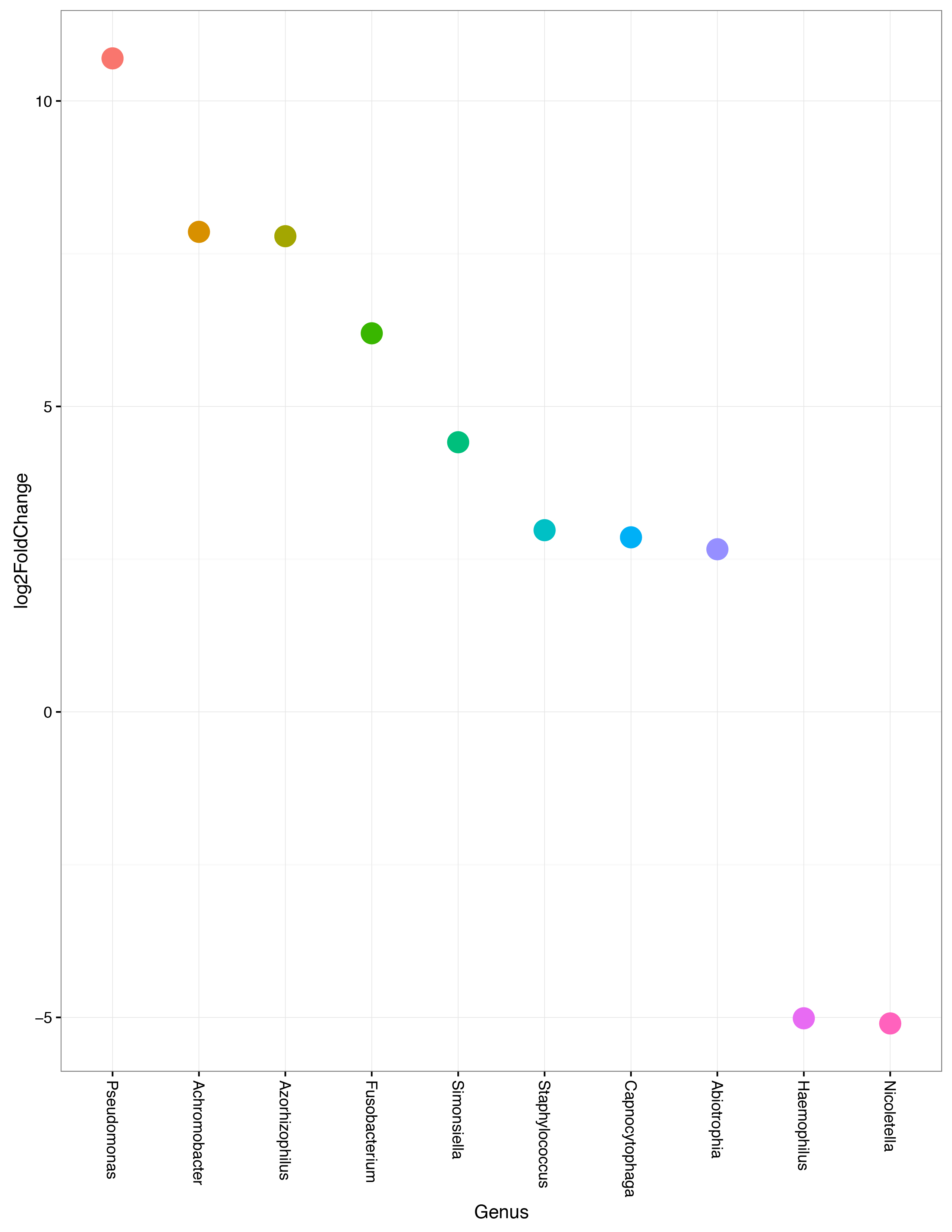


**Figure S4. Ten significant genus-level differences were found in samples obtained from individuals with culture positive sputum for *P. aeruginosa*.** Genera were deemed significant using DESeq2. The change in log_2_ fold abundance of each genus was deemed significant if α<0.01. OTUs were grouped into genera prior to Beta-diversity analysis.


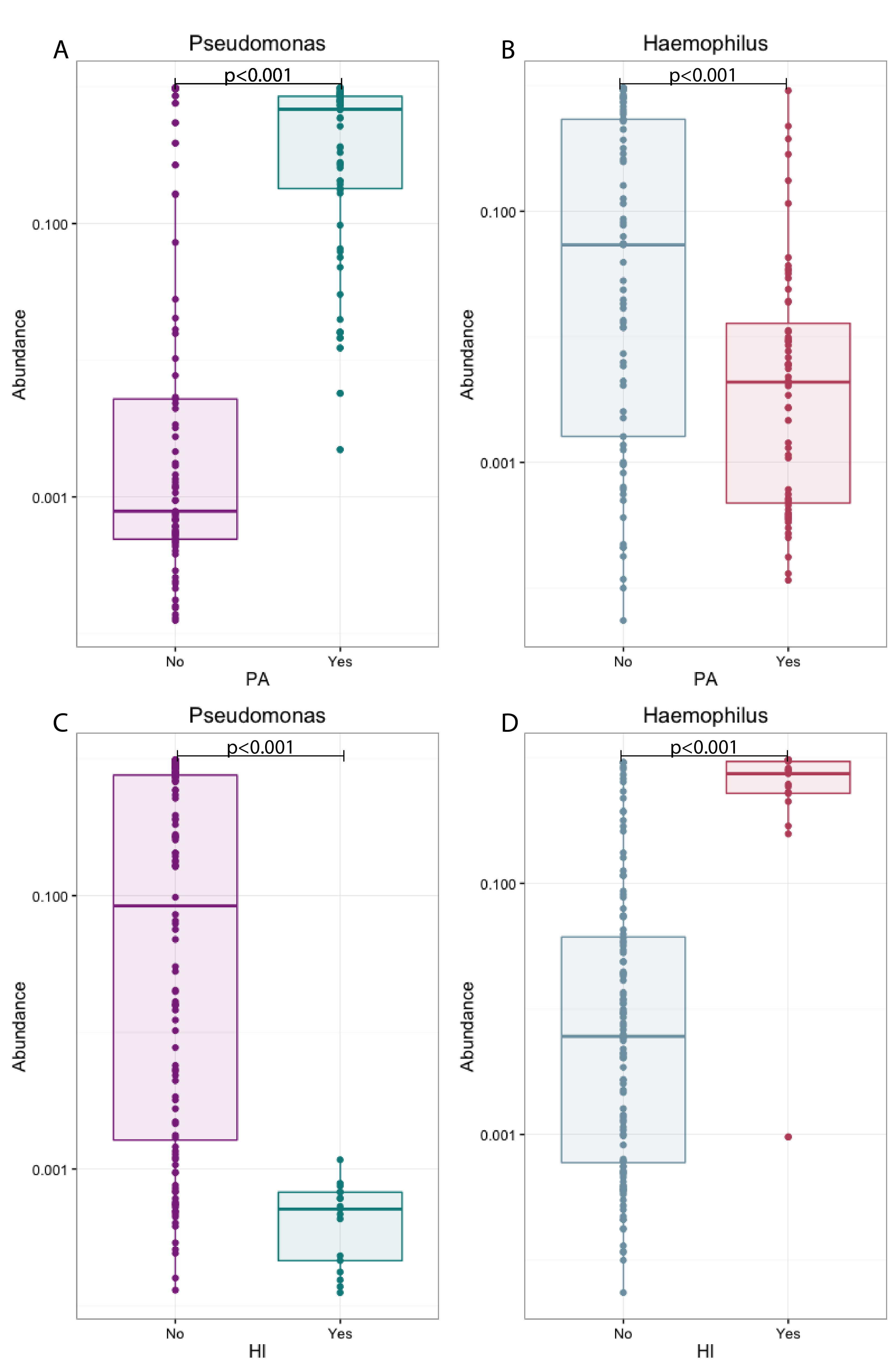


**Figure S5.** **The abundance of *Pseudomonas* and *Haemophilus* was measured in samples where *P. aeruginosa* (PA) or *H. influenzae* (HI) was isolated (Yes) using standard culture methods and compared to the median abundance of samples where *P. aeruginosa* or *H. influenzae* were not isolated (No).** Abundance of *Pseudomonas* (A) and *Haemophilus* (B) was measured in samples coinciding with the isolation *of P. aeruginosa* in culture (n=60). Similarly, the abundance of *Pseudomonas* (C) and *Haemophilus* (D) was measured for samples collected with *H. influenzae* in sputum culture (n=17). The median and interquartile ranges (IQR) are represented by the middle, top, and bottom lines of each boxplot. A log10 y-axis was used to more accurately represent the abundance of each group. The Wilcoxon signed rank test was used to determine significance; medians of each group were deemed significant if p<0.05. The *** symbol is representative of a p<0.001.


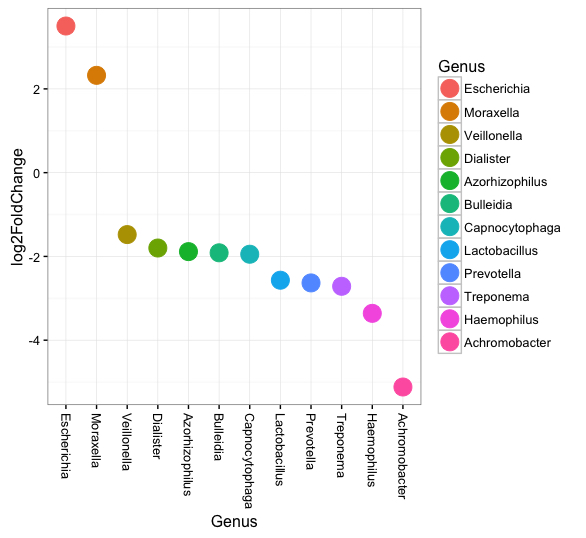


**Figure S6. Twelve significant genus-level differences were observed in samples obtained during fluoroquinolone use.** Genera were deemed significant using DESeq2. The change in log_2_ fold abundance of each genus was deemed significant if α<0.01. OTUs were grouped into genera prior to Beta-diversity analysis.
